# Evaluation of the Efficacy of Tenofovir Alafenamide in Patients with Low-Level Viremia Under Chronic Hepatitis B Treatment

**DOI:** 10.3390/v17111471

**Published:** 2025-11-04

**Authors:** Alper Tahmaz, Merve Yıldız Dikmen, Figen Yıldırım, Türkkan Öztürk Kaygusuz, Oğuz Karabay, Mustafa Kemal Çelen, Sevil Alkan, Tuba Damar Çakırca, Fethiye Akgül, Sıla Akhan, Esra Gürbüz, Şafak Özer Balin, Mehmet Çelik, Mehmet Çabalak

**Affiliations:** 1 Clinic of Infectious Diseases and Clinical Microbiology, Antalya Training and Research Hospital, Antalya 07000, Türkiye; e.merve.yildiz.96@gmail.com; 2 Clinic of Infectious Diseases and Clinical Microbiology, Antalya Life Hospital, Antalya 07000, Türkiye; drfigensarigul@yahoo.com.tr; 3 Department of Infectious Diseases and Clinical Microbiology, Faculty of Medicine, Fırat University, Elazıg 23000, Türkiye; turkkan69@gmail.com (T.Ö.K.); safakozerbalin@hotmail.com (Ş.Ö.B.); 4 Department of Infectious Diseases and Clinical Microbiology, Faculty of Medicine, Sakarya University, Sakarya 54000, Türkiye; oguzkarabay54@gmail.com; 5 Department of Infectious Diseases and Clinical Microbiology, Faculty of Medicine, Dicle University, Diyarbakır 21000, Türkiye; mkcelen@hotmail.com; 6 Department of Infectious Diseases and Clinical Microbiology, Faculty of Medicine, Çanakkale On Sekiz Mart University, Çanakkale 17000, Türkiye; sevil3910@gmail.com; 7 Clinic of Infectious Diseases and Clinical Microbiology, Şanlıurfa Training and Research Hospital, Şanlıurfa 63000, Türkiye; dr.tubadamar@gmail.com; 8 Clinic of Infectious Diseases and Clinical Microbiology, Batman Training and Research Hospital, Batman 72000, Türkiye; dr.fethiyeakgul@gmail.com; 9 Department of Infectious Diseases and Clinical Microbiology, Faculty of Medicine, Kocaeli University, Kocaeli 41000, Türkiye; sila.akhan@kocaeli.edu.tr; 10 Clinic of Infectious Diseases and Clinical Microbiology, Van Training and Research Hospital, Van 65000, Türkiye; dr.inanhazan@gmail.com; 11 Department of Infectious Diseases and Clinical Microbiology, Faculty of Medicine, Harran University, Şanlıurfa 63000, Türkiye; dr.mcelik12@gmail.com; 12 Department of Infectious Diseases and Clinical Microbiology, University Faculty of Medicine, Hatay Mustafa Kemal Üniversitesi, Hatay 31000, Türkiye; mehcab@yahoo.com

**Keywords:** low level viremi, Hepatitis B, tenofovir, tenofovir alafenamide (TAF), entecavir, efficiency, hepatocellular carcinoma

## Abstract

In this multicenter, retrospective study involving 62 patients, we investigated whether switching from entecavir (ETV) or tenofovir disoproxil fumarate (TDF) to tenofovir alafenamide (TAF) represents a superior treatment strategy for patients with chronic hepatitis B (CHB) experiencing low-level viremia (LLV). The study determined that TAF significantly improved both virological and biochemical outcomes. At 48 weeks, the complete virological response (CVR) rate was 77.8% for those who switched from ETV and 81.8% for those who switched from TDF, with Hepatitis B virus deoxyribonucleic acid (HBV DNA) negativity reaching 81% by month 12. Additionally, significant normalization of liver enzymes, albumin, and platelet counts was observed across the cohort. While the switch from TDF was associated with a significant increase in triglycerides and high-density lipoprotein (HDL) and a decrease in estimated glomerular filtration rate (eGFR), no such changes were detected in the ETV group. This evidence suggests that TAF provides robust virological control in LLV patients and is associated with favorable biochemical improvements. However, due to the study’s limitations, the strong assertion that TAF promotes the regression of liver fibrosis and reduces the risk of hepatocellular carcinoma (HCC) must be interpreted with caution.

## 1. Introduction

Hepatitis B virus (HBV) is an important human pathogen, characterized as a hepatotropic DNA virus that primarily targets hepatocytes and is the causative agent of a wide spectrum of liver diseases [1]. Although the widespread introduction of universal neonatal vaccination has significantly curtailed the incidence of new HBV infections globally, an estimated 257 million people worldwide are currently living with chronic HBV infection, and approximately 25% of these individuals succumb to complications such as hepatocellular carcinoma or liver failure [2]. Chronic hepatitis B (CHB) remains the primary driver of liver-related morbidity and mortality globally. A deeper understanding of the natural history of HBV infection has been crucial, leading to significant advances in the development of antiviral therapies and the clinical management of patients with CHB. Effective antiviral treatment relies on potent nucleoside/nucleotide analogue (NNA) drugs, such as ETV, TDF, and TAF, which possess high genetic barriers. Treatment with these agents has been consistently demonstrated to promote the regression of liver fibrosis, prevent liver-related complications, and ultimately improve patient survival [3,4,5,6]. Nevertheless, despite the use of potent antiviral agents, the risk of developing hepatic complications, particularly HCC, has not been completely eradicated in patients with CHB [6,7]. LLV may be a possible cause of HCC in NNA-treated patients [6,8,9]. LLV is not mentioned in most guidelines, with the exception of the American Association for the Study of Liver Diseases (AASLD) guideline (2018). In this guideline, LLV is defined as detectable HBV DNA < 2000 IU/mL (detection limit 10 IU/mL) after 48 weeks of antiviral treatment [10]. If a patient develops LLV, it has not yet been determined whether the patient should continue their original treatment regimen or switch to alternative therapies. Guidelines recommend that patients exhibiting a partial virological response to non-first-line agents should be switched to the most effective antiviral agent lacking cross-resistance [6,10,11,12,13]. For patients with LLV, the AASLD recommends that those currently receiving ETV or TDF monotherapy should continue their existing monotherapy regimen; however, the quality and certainty of this evidence are low [10]. The European Association for the Study of the Liver (EASL) does not recommend altering the initial treatment strategy in patients on potent NNA monotherapy who exhibit low HBV DNA levels (HBV DNA < 69 IU/mL) and/or decreasing HBV DNA concentrations. Conversely, if the HBV DNA level has plateaued (69 < DNA < 2000 IU/mL), switching to an alternative agent or combining ETV with TDF or TAF should be considered [12]. Recent studies have demonstrated that switching from ETV to TAF facilitates the achievement of undetectable HBV DNA at some point following the switch in patients with LLV [14,15,16]. However, it remains unclear whether switching from ETV to TAF can achieve and sustain a CVR during TAF treatment in patients with LLV or occasionally detectable HBV DNA. Accordingly, the primary objective of this study was to investigate the effectiveness of switching from ETV and TDF to TAF in achieving CVR in patients with LLV. Although most CHB patients treated with NNA are well-controlled, long-term NNA therapy carries a notable risk of nephrotoxicity and bone toxicity. TAF possesses high plasma stability, enabling the efficient delivery of the active metabolite to hepatocytes at a low 25 mg dose, resulting in reduced circulating tenofovir levels and minimizing the risk of long-term renal and bone damage [17,18,19]. However, studies have demonstrated that TAF performs worse than TDF concerning changes in fasting lipid profiles, and the exact underlying mechanism of these alterations remains to be fully elucidated [20]. Our secondary aim was to evaluate the virological, biochemical, and renal safety outcomes following the switch from ETV or TDF to TAF.

## 2. Materials and Methods

The records of patients attending Infectious Diseases and Clinical Microbiology outpatient clinics at participating centers between January 2018 and January 2024 were retrospectively reviewed. The analysis focused on patients ≥ 18 years of age who had been receiving either ETV or TDF monotherapy for CHB infection for a minimum of 48 weeks, but who had failed to achieve a CVR. Specifically, we identified patients followed with LLV, defined as an HBV DNA level between 10−2000 IU/mL measured at 12-week intervals despite 48 weeks of effective treatment [10]. Patients were included if they met the LLV criteria, had their treatment subsequently switched to TAF during follow-up, and continued TAF therapy for at least 48 weeks.

Exclusion criteria applied to patients were: those who failed to achieve CVR but did not meet the LLV definition (i.e., had higher HBV DNA levels); those switched to TAF due to concerns over renal/bone safety or side effects while on TDF or ETV; and patients who had received ETV and TDF combination therapy prior to the TAF switch. Furthermore, patients with a history of interferon treatment; coinfection with Hepatitis C Virus or Human Immunodeficiency Virus; other underlying liver diseases, including autoimmune liver disease or drug-induced liver injury; with cirrhosis or advanced chronic liver disease; any history of malignancy; poor medication adherence; or who were pregnant or breastfeeding were excluded from the study.

### 2.1. Safety and Efficacy Evaluation

The safety profile and virological efficacy of switching from current TDF or ETV to TAF were critically evaluated over a 12-month follow-up period. Patient safety and treatment efficacy were meticulously assessed through comprehensive clinical examinations and laboratory investigations at baseline (during the switch to TAF) and at weeks 12, 24, and 48 following the switch to TAF.

1. Virologic response and treatment efficacy, particularly in the context of LLV, were monitored through:

HBV DNA Quantification: Serum HBV DNA levels were measured using a high-sensitivity Real-Time Polymerase Chain Reaction assay (Lower Limit of Detection: [Serum HBV DNA levels were measured using the COBAS TaqMan HBV Test (Roche, Branchburg, NJ 08876-3771, USA), with a lower limit of detection of 10 IU/mL.]).

Hepatitis B e-Antigen (HBeAg) Status: HBeAg and Hepatitis B e-antibody (anti-HBe) antibody status were determined to track potential seroconversion or seroreversion events.

Hepatitis B surface antigen (HBsAg): HBsAg levels were periodically monitored to assess the possibility of functional cure. HBV serological markers were measured by chemiluminescent immunoassay (Abbott Diagnostics, Wiesbaden, Germany).

2. Renal Safety:

Serum creatinine, calculated eGFR using the Cockcroft-Gault method, and serum phosphate levels were assessed to detect potential changes in renal tubular or glomerular function, which is a key safety outcome following the switch from TDF.

3. Hepatic Function:

Liver biochemistries, including alanine aminotransferase (ALT), aspartate aminotransferase (AST), Total Bilirubin, and Albumin, were monitored to track any drug-induced hepatotoxicity or unexpected flare events. Aspartate aminotransferase platelet ratio index (APRI) scores were calculated for all patients using the formula APRI = ([AST/ULN]/PLT) × 100 [17].

### 2.2. Adverse Events and Clinical Outcomes

The primary safety analysis focused on the incidence, severity, and clinical impact of Adverse events and serious adverse events following the TAF switch.

The primary efficacy endpoint was the maintenance of virologic suppression (HBV DNA < 10 IU/mL) throughout the 12-month follow-up. The secondary efficacy endpoints include the degree of reduction in serum HBV DNA levels among patients with detectable HBV DNA; ALT normalization; HBeAg seroconversion; and changes in liver fibrosis as measured by the APRI (AST to Platelet Ratio Index) [17].

Patient data collected in the study were analyzed using IBM Statistical Package for the Social Sciences (SPSS) 30.0 for Macos 30.0 (IBM Corp., Armonk, NY, USA). Descriptive values included frequencies and percentages for categorical data and medians, 25% and 75% quartiles for continuous data. Chi-square or Fisher’s exact test was used to compare categorical variables. Friedman’s two-way analysis of variance with ranks was used to assess differences between dependent measures. For pairwise comparisons between dependent measures, multiple testing was evaluated with Bonferroni correction. Results were considered statistically significant if the *p*-value was less than 0.05.

### 2.3. Definition

Chronic Hepatitis B: Persistence of HBsAg for 6 months or more after acute infection with hepatitis B virus [21].

HBsAg: A viral protein detectable in the blood in acute and chronic hepatitis B infection [21].

HBsAg seroconversion: The development of antibodies against HBsAg is known as HBsAg seroconversion. It signifies clearance of HBsAg and resolution of the chronic infection [21].

HBV DNA (Viral Load): The HBV DNA level, or ‘viral load,’ is an indicator of viral replication [21].

HBeAg Seroconversion: Occurs when people infected with the HBeAg-positive form of the virus develop antibodies against the ‘e’ antigen (UK, National Clinical Guideline Centre [21]).

Low-Level Viremia: Defined as the persistent maintenance of HBV DNA levels between 10 and 2000 IU/mL after 48 weeks of follow-up despite ongoing effective antiviral treatment, indicating a failure to achieve a complete virological response [10].

Complete Virological Response: Defined as persistently undetectable HBV-DNA (by a sensitive polymerase chain reaction assay) for NNA-based treatment, or HBV-DNA < 2000 IU/mL after therapy discontinuation in Interferon-α regimens [12].

Cirrhosis: An irreversible, advanced-stage liver disease caused by the HBV. The diagnosis is established through a combination of clinical findings, laboratory tests, and imaging modalities. Laboratory indicators include thrombocytopenia (platelet count < 150,000/μL), hypoalbuminemia (albumin level < 3.5 g/dL), a prolonged International Normalized Ratio (INR > 1.2), elevated bilirubin, and an APRI score > 2. Imaging studies such as ultrasound, computed tomography, or magnetic resonance imaging typically reveal a nodular liver contour, splenomegaly, and signs of portal hypertension (e.g., varices). The gold standard for definitive diagnosis remains a liver biopsy, which confirms the presence of stage 4 fibrosis or established cirrhosis upon histological examination [10,12,22].

## 3. Results

In our study, a total of 62 patients who fulfilled the inclusion and exclusion criteria were evaluated. Of these patients, 67.7% (*n* = 42) were male. Patient age ranged from 26 to 77 years, with a median age of 43 years. Prior to switching to TAF treatment, 29% (*n* = 18) of the patients were receiving ETV, and 71% (*n* = 44) were receiving TDF. Upon grouping patients according to their baseline HBV DNA levels, the largest proportion of the cohort (43.5%) fell within the 100−1000 IU/mL range. Twelve patients were HBeAg-positive. All patients exhibited liver, renal, and metabolic test results that were within the normal reference ranges. During the entire 12-month follow-up period, no adverse events, including unexpected serious adverse events, were observed or reported in any patient following the switch to TAF. The cohort consistently maintained clinical stability, with no incidence of clinical decompensation, liver-related hospitalization, or all-cause mortality. Consequently, given the favorable safety profile and the durable maintenance of virologic suppression, no patients necessitated dose adjustment, temporary interruption, or permanent discontinuation of TAF therapy attributable to adverse events or laboratory abnormalities throughout the 12-month study (Table 1).

The patient data, including outcomes for low-level viremia, before and after TAF treatment at three, six, and twelve months, are detailed in Table 2. Following the initiation of TAF therapy, HBV DNA negativity was achieved in 67.3% (*n* = 37), 67.9% (*n* = 38), and 81% (*n* = 50) of patients at three, six, and twelve months, respectively. When classified according to baseline HBV DNA levels, it was observed that in 74.5% of patients, HBV DNA dropped below <20 IU/mL starting from the 3rd month of TAF treatment. At the end of the 12th month of TAF treatment, 87.1% of patients’ HBV DNA was below <20 IU/mL. No statistically significant difference in HBeAg seroconversion was observed at any point following the treatment modification. Furthermore, while HBeAg positivity was seen to recur in one patient who switched from TDF to TAF, this occurrence did not reach statistical significance. Liver enzymes (ALT and AST), albumin, and platelet counts were significantly normalized with TAF treatment. Although alpha-fetoprotein (AFP) and the APRI score—both indicators of liver damage—normalized with the treatment change, these results were not statistically significant. HDL cholesterol levels significantly improved, whereas no difference was observed in low-density lipoprotein (LDL) cholesterol. Conversely, triglyceride levels significantly increased beginning a few months after switching to TAF. The eGFR values, which served to monitor changes in patients’ renal function, significantly decreased during the follow-up period (Table 2).

When the data from patients who switched from ETV and TDF to TAF were analyzed individually (Table 3 and Table 4), HBV DNA negativity was achieved in 77.8% and 81.8% of the LLV patients in the ETV and TDF groups, respectively, at the end of the twelfth month. ALT normalization was significantly observed after switching from both drugs to TAF. Although no statistically significant differences were observed in comparisons analyzing changes in drug levels, a significant increase in triglycerides and HDL was detected in the TDF group following the switch to TAF, whereas no such change was observed in the ETV group. Similarly, while eGFR significantly decreased in the TDF group after the switch to TAF, no corresponding change was noted in the ETV group.

## 4. Discussion

Low-level viremia is considered a potential risk factor for the development of HCC in patients receiving NNA treatment [6,8,9]. Therefore, effective management of LLV and ensuring a complete CVR are crucial steps toward mitigating the risk of HCC. When LLV develops, clinical consensus is lacking on whether monotherapy should be continued or if a switch to an alternative agent is warranted. Current guidelines advocate that patients with a partial virological response to non-first-line agents should transition to the most potent antiviral agent that lacks cross-resistance [6,10,12,13]. We analyzed the efficacy and safety of TAF monotherapy as an alternative treatment for patients who developed LLV while receiving ETV, TDF, or combination therapies. In the literature, He et al. reported that among a group of 351 LLV patients, the complete CVR rate after 48 weeks was 75.3% in the TAF group (*n* = 183) compared to 11.4% in the ETV group (*p* < 0.001) [17]. Similarly, Zhong et al., in a study encompassing 211 LLV patients, demonstrated that the CVR rate was 62% in the TAF group versus 9% in the ETV group, concluding that the CVR rate was significantly higher in the TAF group [14]. Ogawa et al. studied 313 consecutive CHB patients who were treated with ETV or a NNA combination regimen for more than two years and subsequently switched to TAF monotherapy, achieving a CVR of 73.9% in the ETV group and 77.8% in the NNA combination group [15]. The fact that we achieved a CVR of 81% (*n* = 50) among our 62 patients—who had been followed for a median of 44 months despite receiving ETV and TDF treatment—48 weeks after switching to TAF is consistent with the published literature. The achievement of CVR is critical for long-term clinical benefit and was therefore designated as the primary endpoint of our study.

In terms of drug-related factors, TAF’s high potency, liver-targeting properties, and notable plasma stability lead to a lower potential for side effects, providing the theoretical basis for the superior response observed in the TAF group [14]. Regarding LLV specifically, Zhong et al. demonstrated ALT normalization in 47% of the TAF group versus 10% in the ETV group at 24 weeks, and this difference was statistically significant in favor of TAF [14]. Similarly, He et al. compared patients receiving TAF and ETV for low viremia and reported that ALT normalization was significantly higher in the TAF arm [17]. Our findings align with the literature; ALT normalization after switching to TAF treatment was found to be significant in patients receiving long-term ETV or TDF but progressing to LLV. ALT normalization was significantly observed in patients who switched from both TDF and ETV to TAF. This difference is likely attributable to the superior viral suppression achieved in the TAF group.

Both the APRI and the Fibrosis-4 index (FIB-4) are recommended by WHO guidelines for the clinical assessment of liver fibrosis [23]. We utilized APRI to evaluate liver fibrosis due to its ease of use, non-invasiveness, and quantitative nature. Our results demonstrated no statistically significant change in APRI scores after 48 weeks of TAF treatment in patients with LLV. The likely reason for this finding is that the patients had previously received ETV or TDF therapy for an average of 44 months. This prior treatment led to liver improvement in the majority of the cohort before TAF initiation. Consequently, achieving a significant further reduction after only 48 weeks of follow-up is challenging, necessitating studies with longer follow-up durations to observe this difference.

TDF is actively secreted by the kidneys via organic anion transporters (OAT1 and OAT3), which leads to high tenofovir exposure in the proximal renal tubules [24,25]. In contrast, TAF is not a substrate of renal OATs and, therefore, does not exhibit OAT-dependent cytotoxicity [25]. Although some publications demonstrate the rapid recovery of lost renal function, evidenced by increased eGFR, after switching from TDF to TAF [26,27], Hishijima et al. found that long-term TDF use and subsequent discontinuation, particularly when utilized for more than two years, significantly increased the likelihood of causing renal tubular damage compared to those who had never used TDF. Furthermore, they showed that cumulative TDF exposure, independent of current TDF use, was strongly and robustly associated with renal damage [28]. Two studies analyzing the change in eGFR over time following the switch from TDF to TAF demonstrated a significant decrease in eGFR in patient groups with an initial eGFR ≥ 90 mL/min/1.73 m^2^ at 144 weeks and 18 months, respectively; conversely, increases in eGFR were observed in the lower eGFR groups after the switch [29,30]. In our study, the median duration of TDF treatment prior to the switch to TAF was 44 months. The decline in eGFR continued during follow-up in our patient population, which primarily consisted of individuals with high cumulative TDF exposure and an eGFR ≥ 90 mL/min/1.73 m^2^. This finding supports the existing literature in two aspects concerning the deterioration of renal function in patients who continued TAF after TDF. Tsai et al. showed that eGFR remained stable in the ETV group during the first three years and subsequently improved significantly in years four and five [31]. Consistent with this finding, our study demonstrated no change in eGFR in those who switched from ETV to TAF.

In terms of lipid safety, TDF treatment is widely known to exert a ‘lipid-lowering effect’ due to high plasma tenofovir concentrations. Clinical trials have reported that patients who switched from TDF to TAF exhibited a greater increase in triglycerides, LDL, and HDL cholesterol compared to those who continued TDF therapy, a finding associated with the lipid reduction observed in TDF-receiving patients [17,32,33]. Over forty-eight weeks, no significant increases in HDL, LDL, or triglycerides were observed in the group that switched from ETV to TAF. Specifically, no significant changes were detected in the ETV-to-TAF group compared to baseline. In contrast, the group that switched from TDF to TAF showed a significant increase in HDL and triglycerides relative to baseline, while no significant change was noted in LDL. The finding that no change in lipid levels was observed in the ETV-to-TAF group, and the increase in lipid parameters in the TDF-to-TAF group, collectively support the lipid-lowering effect of TDF. This implies that TAF exhibits an effect consistent with ‘normal blood lipid levels’ when compared to TDF. However, the clinical significance of TAF’s lipid effect remains unclear, and a definitive mechanism for these observed changes has yet to be identified [17]. The metabolic effects of antiviral drugs on patients should not be underestimated. Monitoring of blood lipid parameters is thus recommended for the long-term follow-up of TAF treatment. Future studies should focus on the actual cardiovascular risk rather than solely on changes in lipid parameters [17]. Considering that most CHB patients require lifelong treatment and that the prevalence of comorbidities increases with age, changes in both renal function and lipid parameters necessitate rigorous monitoring and management.

One of the limitations of our study is the relatively small number of patients, particularly in the ETV group, despite the multicenter design. Although non-invasive techniques were utilized for the evaluation of hepatic fibrosis, these methods only provide indirect assessments and cannot furnish the definitive histological evidence that a liver biopsy offers. Moreover, the 48-week observation period may be insufficient to adequately evaluate challenging clinical endpoints, such as cirrhosis progression and the incidence of HCC. HBV genotype has been shown to be a crucial factor affecting treatment efficacy, as demonstrated by Uchida et al. [34]. While genotypic analysis could potentially offer more precise guidance for rescue therapies in LLV patients, the absence of HBV genotype information constituted another limitation of our study. Although HBV genotypes are generally not thought to significantly influence initial virologic responses to NNA, the role of genotype may become more pronounced during long-term viral suppression failure and in the context of LLV. One prominent cause of LLV is the emergence of mutations that impair the virus’s ability to produce HBeAg (pre-core/basal core promoter mutants). These HBeAg-negative mutant strains can more readily evade the host immune system and persist at low levels despite NNA suppression, consequently leading to LLV. Genotypes with a high propensity for mutation, such as Genotypes C and D, may drive the formation of these HBeAg-negative mutant strains, thereby hindering complete viral suppression and increasing the risk of LLV development [35,36]. This highlights the critical importance of genotype analysis for managing patients with LLV, particularly in regions like Turkey where Genotype D is highly prevalent [37,38,39]. Consequently, future studies utilizing larger sample sizes and advanced genotyping techniques are essential to better elucidate the precise role of HBV genotype in patients experiencing LLV. However, an important limitation of our retrospective study, based on patient chart review, was the incomplete capture of anthropometric data. Specifically, body weight measurements were either missing or insufficient for a substantial proportion of patients. Consequently, we were unable to provide additional analysis or definitive conclusions regarding the influence of body mass index (BMI) or weight changes on treatment outcomes following the switch to TAF. This data gap restricts our ability to fully evaluate the metabolic safety and efficacy relationship in our cohort. The increase in BMI after switching to TAF is associated with a decrease in eGFR, but we could not analyze the effect of BMI on the decrease in eGFR in patients switching to TAF after TDF because we did not have data on this.

## 5. Conclusions

In conclusion, switching to TAF therapy may be a favorable option for patients who develop low-level viremia during ETV or TDF monotherapy. There are several justifications for this: TAF provides superior virologic and biochemical responses and its safety profile is comparable to that of ETV and TDF. Larger cohort, comprehensive, and long-term studies are needed to evaluate the effect of TAF on the pathological improvement of the liver following treatment change in patients with LLV.

## Figures and Tables

**Table 1:** Distribution of patient demographics and pre-treatment laboratory findings.

Variables	*n* (%) or Median (IQR)
Age (years)	43 (36–54)
Gender	
Female	20 (32.3)
Male	42 (67.7)
Pre-TAF treatment	
ETV	18 (29)
TDF	44 (71)
Duration of treatment before TAF (months)	44 (30–80)
Pre-treatment HBV DNA level (IU/mL)	
<20	9 (14.5)
21–100	15 (24.2)
101–1000	27 (43.5)
>1000	11 (17.7)
HBeAg (+)	12 (19.4)
Anti-HBe (+)	50 (80.6)
AST (U/L)	29 (21–41)
ALT (U/L)	34 (20–45)
Albumin (g/L)	4 (3.8–4.5)
Total Bilirubin (mg/dL)	0.7 (0.5–0.9)
AFP (μg/L)	2.8 (1.8–3.2)
HDL (mg/dL)	43 (40–46)
LDL (mg/dL)	114 (99–126)
Total Cholesterol (mg/dL)	207 (199–209)
Triglyceride (mg/dL)	189 (166–208)
Platelet count (10^3^/μL)	237.5 (210–302)
eGFR (mL/dk/1.73m^2^)	94 (88–104)
Phosphorus (mg/dL)	3 (2.9–3.3)
APRI	0.4 (0.2–0.8)
HBsAg seroconversion	0
Adverse events	0
Mortality	0

Abbreviations: AFP, alpha-fetoprotein; ALT, alanine aminotransferase; APRI, aspartate aminotransferase platelet ratio index; AST, aspartate aminotransferase; eGFR, estimated glomerular filtration rate; Anti-HBe, Hepatitis B virus e antibody; HBeAg, Hepatitis B virus e antigen; HBV-DNA, Hepatitis B virus deoxyribonucleic acid; HDL, High density lipoprotein; LDL, Low density lipoprotein; TAF, Tenofovir alafenamide.

**Table 2:** Distribution of patients’ laboratory values before and after treatment.

Variables	Pre-TAF ^1^ (*n* = 62)	TAF 3rd Month ^2^ (*n* = 55)	TAF 6th Month ^3^ (*n* = 56)	TAF 12th Month ^4^ (*n* = 62)	*p*-Value	Post-HOC
	*n* (%) or Median (IQR)	*n* (%) or Median (IQR)	*n* (%) or Median (IQR)	*n* (%) or Median (IQR)		
HBV-DNA (log10)	2.46 (1.48–2.83)	1.83 (1.3–3.02)	1.46 (1.14–1.81)	1.6 (1.12–2.22)	0.143	
HBV-DNA (+)	62 (100)	18 (32.7)	18 (32.1)	12 (19.4)	<0.001	
HBV-DNA (IU/mL)					<0.001	1–2;2–4;3–4
<20	9 (14.5)	41 (74.5)	45 (80.4)	54 (87.1)		
20–100	15 (24.2)	6 (10.9)	8 (14.3)	4 (6.5)		
100–1000	27 (43.5)	4 (7.3)	2 (3.6)	3 (4.8)		
>1000	11 (17.7)	4 (7.3)	1 (1.8)	1 (1.6)		
HBeAg (+)	12 (19.4)	11 (20)	11 (19.6)	13 (21)	0.996	
Anti-HBe (+)	50 (80.6)	44 (80)	45 (80.4)	49 (79)	0.996	
AST (U/L)	29 (21–41)	26 (21–30)	27.5 (21.5–33)	27.5 (21–33)	0.046	1–2;1–4
ALT (U/L)	34 (20–45)	26 (20–33)	28 (18–41.5)	28 (18–36)	0.002	1–2;1–3;1–4
Albumin (g/L)	4 (3.8–4.5)	4 (3.6–4.6)	4.1 (3.6–4.4)	4.3 (3.7–4.6)	0.004	1–4;2–4;3–4
Total Bilirubin (mg/dL)	0.7 (0.5–0.9)	0.7 (0.5–1)	0.8 (0.5–0.9)	0.8 (0.5–1)	0.598	
AFP (μg/L)	2.8 (1.8–3.2)	2.5 (2–3.7)	2.6 (1.8–3.9)	2.5 (2–3.7)	0.477	
HDL (mg/dL)	43 (40–46)	44 (41–47)	44 (41–51)	46 (41–52)	0.032	1–4;2–4
LDL (mg/dL)	114 (99–126)	109 (96–125)	111 (93.5–130)	102 (88–125)	0.233	
Total Cholesterol (mg/dL)	207 (199–209)	205 (188–217)	201 (183–215)	203 (196–211)	0.475	
Triglyceride (mg/dL)	189 (166–208)	199 (163–211)	200 (168–217)	201 (174–222)	<0.001	1–2;1–3; 1–4;2–4
Platelet count (10^3^/μL)	237.5 (210–302)	241 (194–282)	244.5 (192.5–290.5)	252.5 (195–302)	0.040	1–2;2–4
eGFR (mL/dk/1.73m^2^)	94 (88–104)	93 (88–101)	90 (85.5–99)	90 (87–97)	0.016	1–3;1–4
Phosphorus (mg/dL)	3 (2.9–3.3)	3 (2.8–3.5)	3 (2.9–3.5)	3 (2.8–3.4)	0.724	
APRI	0.4 (0.2–0.8)	0.3 (0.2–0.9)	0.4 (0.2–0.9)	0.3 (0.2–0.9)	0.921	

Abbreviations: AFP, alpha-fetoprotein; ALT, alanine aminotransferase; APRI, aspartate aminotransferase platelet ratio index; AST, aspartate aminotransferase; eGFR, estimated glomerular filtration rate; Anti-HBe, Hepatitis B virus e antibody; HBeAg, Hepatitis B virus e antigen; HBV-DNA, Hepatitis B virus deoxyribonucleic acid; HDL, High density lipoprotein; LDL, Low density lipoprotein; TAF, Tenofovir alafenamide.

**Table 3:** Distribution of laboratory measurements in patients treated with ETV before and after treatment.

Variables	Pre-TAF ^1^ (*n* = 18)	TAF 3rd Month ^2^ (*n* = 17)	TAF 6th Month ^3^ (*n* = 15)	TAF 12th Month ^4^ (*n* = 18)	*p*-Value	Post-HOC
	*n* (%) or Median (IQR)	*n* (%) or Median (IQR)	*n* (%) or Median (IQR)	*n* (%) or Median (IQR)		
HBV-DNA (log10)	1.9 (1.8–2)	1.9 (1.8–2)	1.3 (1.2–1.9)	1.9 (1.5–2.8)	0.122	
HBV-DNA (+)	18 (100)	5 (29.4)	7 (46.7)	4 (22.2)	<0.001	
HBeAg (+)	6 (33.3)	5 (29.4)	5 (33.3)	6 (33.3)	0.993	
Anti-HBe (+)	12 (66.7)	12 (70.6)	10 (66.7)	12 (66.7)	0.993	
AST (U/L)	24 (22–41)	23 (19–30)	27 (19–36)	27 (19–28)	0.028	1–4;3–4
ALT (U/L)	29.5 (19–56)	25 (23–31)	26 (17–45)	26 (14–45)	0.026	1–2;1–3;1–4
Albumin (g/L)	4.5 (3.9–5.1)	4.3 (4.1–4.9)	4.4 (4.2–4.6)	4.5 (4.3–5.1)	0.155	
Total Bilirubin (mg/dL)	0.6 (0.5–0.6)	0.6 (0.5–0.7)	0.6 (0.5–0.8)	0.7 (0.5–0.9)	0.119	
AFP (μg/L)	2 (1.5–2.8)	1.8 (1.7–2.8)	1.8 (1.7–2.6)	2.1 (1.7–3)	0.735	
HDL (mg/dL)	41 (39–45)	42 (40–45)	44 (39–51)	42.5 (41–46)	0.419	
LDL (mg/dL)	109.5 (90–126)	113 (89–120)	111 (90–125)	110 (88–126)	0.191	
Total Cholesterol (mg/dL)	208.5 (201–219)	211 (180–233)	211 (165–233)	209 (201–233)	0.920	
Triglyceride (mg/dL)	183 (152.9–211)	180 (163–201)	176 (92–206)	194.5 (172–211)	0.095	
Platelet count (10^3^/μL)	253.5 (210–302)	250 (205–308)	262 (222–301)	268 (227–310)	0.241	
eGFR (mL/dk/1.73m^2^)	88 (84–104)	92 (88–102)	90 (75–109)	89.5 (74–97)	0.688	
Phosphorus (mg/dL)	3 (2.9–3.9)	3.1 (3–4)	3.2 (3–3.9)	3 (2.7–3.7)	0.197	
APRI	0.2 (0.2–0.4)	0.2 (0.2–0.3)	0.2 (0.2–0.4)	0.2 (0.2–0.2)	0.377	

Abbreviations: AFP, alpha-fetoprotein; ALT, alanine aminotransferase; APRI, aspartate aminotransferase platelet ratio index; AST, aspartate aminotransferase; eGFR, estimated glomerular filtration rate; ETV, entecavir; Anti-HBe, Hepatitis B virus e antibody; HBeAg, Hepatitis B virus e antigen; HBV-DNA, Hepatitis B virus deoxyribonucleic acid; HDL, High density lipoprotein; LDL, Low density lipoprotein; TAF, Tenofovir alafenamide.

**Table 4:** Distribution of laboratory values in TDF-treated patients before and after treatment.

Variables	Pre-TAF ^1^ (*n* = 44)	TAF 3rd Month ^2^ (*n* = 38)	TAF 6th Month ^3^ (*n* = 41)	TAF 12th Month ^4^ (*n* = 44)	*p*-Value	Post-HOC
	*n* (%) or Median (IQR)	*n* (%) or Median (IQR)	*n* (%) or Median (IQR)	*n* (%) or Median (IQR)		
HBV-DNA (log10)	1.8 (1.2–3)	1.8 (1.2–3)	1.5 (0.8–1.8)	1.5 (1.1–2)	0.469	
HBV-DNA (+)	44 (100)	13 (34.2)	11 (26.8)	8 (18.2)	<0.001	
HBeAg (+)	6 (13.6)	6 (15.8)	6 (14.6)	7 (15.9)	0.990	
Anti-HBe (+)	38 (86.4)	32 (84.2)	35 (85.4)	37 (84.1)	0.990	
AST (U/L)	30 (21–42.5)	26 (22–32)	28 (22–33)	29.5 (21–33.5)	0.224	
ALT (U/L)	34 (21–40)	29 (20–37)	28 (20–40)	27 (18–35.5)	0.046	1–2;1–3;1–4
Albumin (g/L)	3.9 (3.7–4.3)	3.7 (3.5–4.3)	4 (3.6–4.4)	4 (3.6–4.6)	0.030	2–4
Total Bilirubin (mg/dL)	0.8 (0.6–1)	0.8 (0.6–1)	0.8 (0.6–1)	0.8 (0.5–1)	0.608	
AFP (μg/L)	2.8 (2–3.3)	2.9 (2.2–3.9)	2.8 (2–4)	2.7 (2.1–3.8)	0.675	
HDL (mg/dL)	42.5 (41–46)	44 (42–48)	44 (42–51)	46 (42–53.5)	0.034	1–2;1–3;1–4
LDL (mg/dL)	115 (101–126)	108 (99–125)	108 (98–132)	102 (88–125)	0.275	
Total Cholesterol (mg/dL)	204.5 (195–209)	201 (188–211)	200 (188–211)	200 (192.5–208.5)	0.458	
Triglyceride (mg/dL)	196 (170.5–208)	201 (176–212)	202 (186–241)	202.5 (177.5–231.5)	<0.001	1–2;1–3;1–4;2–4
Platelet count (10^3^/μL)	230 (209.5–304.5)	237 (193–277)	241 (187–288)	243 (183–301)	0.098	
eGFR (mL/dk/1.73m^2^)	96 (90–103.5)	93.5 (89–99)	90 (87–98)	90 (88.5–97)	0.006	1–3;1–4
Phosphorus (mg/dL)	3 (2.9–3.3)	3 (2.8–3.2)	3 (2.9–3.3)	3 (2.9–3.4)	0.648	
APRI	0.6 (0.2–0.9)	0.8 (0.3–0.9)	0.7 (0.3–0.9)	0.7 (0.2–1)	0.895	

Abbreviations: AFP, alpha-fetoprotein; ALT, alanine aminotransferase; APRI, aspartate aminotransferase platelet ratio index; AST, aspartate aminotransferase; eGFR, estimated glomerular filtration rate; Anti-HBe, Hepatitis B virus e antibody; HBeAg, Hepatitis B virus e antigen; HBV-DNA, Hepatitis B virus deoxyribonucleic acid; HDL, High density lipoprotein; LDL, Low density lipoprotein; TAF, Tenofovir alafenamide. TDF, tenofovir disoproxil fumarate.

## Data Availability

The Excel and SPSS datasets, as well as the SPSS data outputs related to this study, are kept by the corresponding author. The data presented in this study are available on request from the corresponding author.

## Abbreviations

The following abbreviations are used in this manuscript:

ETV	Entecavir
TDF	Tenofovir disoproxil fumarate
TAF	Tenofovir alafenamide
LLV	Low-level viremia
CVR	Complete virological response
HBV DNA	Hepatitis B virus deoxyribonucleic acid
HDL	High density lipoprotein
eGFR	Estimated glomerular filtration rate
HCC	Hepatocellular carcinoma
HBV	Hepatitis B virus
CHB	Chronic hepatitis B
NNA	Nucleoside/nucleotide analogue
AASLD	American Association for the Study of Liver Diseases
EASL	European Association for the Study of the Liver
HBeAg	Hepatitis B virus e antigen
HBsAg	Hepatitis B surface antigen
ALT	Alanine aminotransferase
AST	Aspartate aminotransferase
APRI	Aspartate aminotransferase platelet ratio index
SPSS	Statistical Package for the Social Sciences
INR	International Normalized Ratio
AntiHBe	Hepatitis B virus e antibody
AFP	Alpha-fetoprotein
LDL	Low density lipoprotein
FIB-4	Fibrosis-4 index
OAT1	Organic anion transporters 1
OAT4	Organic anion transporters 4
BMI	Body mass index
